# Escape motility of multicellular magnetotactic prokaryotes

**DOI:** 10.1098/rsif.2024.0310

**Published:** 2024-10-16

**Authors:** Xinyi Yang, Manu Prakash, Douglas R. Brumley

**Affiliations:** ^1^ School of Mathematics and Statistics, The University of Melbourne, Parkville, Victoria 3010, Australia; ^2^ Department of Bioengineering, Biology and Oceans, Stanford University, Stanford, CA, USA

**Keywords:** magnetotaxis, aerotaxis, cell motility, hydrodynamic interactions, cilia, flagella

## Abstract

Microorganisms often actively respond to multiple external stimuli to navigate toward their preferred niches. For example, unicellular magnetotactic bacteria integrate both oxygen sensory information and the Earth’s geomagnetic field to help them locate anoxic conditions in a process known as magneto-aerotaxis. However, for multicellular magnetotactic prokaryotes (MMPs), the colonial structure of 4–16 cells places fundamental constraints on collective sensing, colony motility and directed swimming. To investigate how colonies navigate environments with multiple stimuli, we performed microfluidic experiments of MMPs with opposing magnetic fields and oxygen gradients. These experiments reveal unusual back-and-forth excursions called ‘escape motility’, in which colonies shuttle along magnetic field lines, punctuated by abrupt—yet highly coordinated—changes in collective ciliary beating. Through cell tracking and numerical simulations, we demonstrate that escape motility can arise through a simple magneto-aerotaxis mechanism, which includes the effect of magnetic torques and chemical sensing. At sufficiently high densities of MMPs, we observe the formation of dynamic crystal structures, whose stability is governed by the magnetic field strength and near-field hydrodynamic interactions. The results shed light on how some of the earliest multicellular organisms navigate complex physico-chemical landscapes.

## 1. Introduction

**Figure 1.**
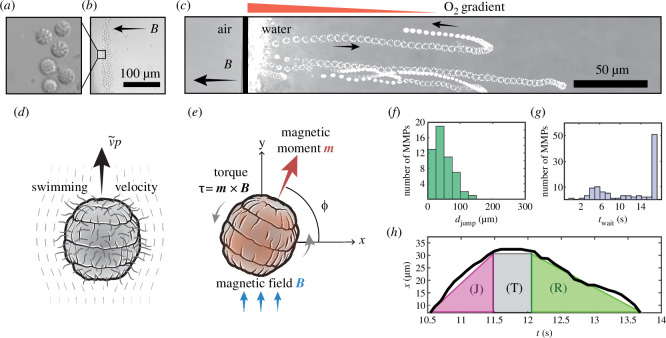
Coordinated reversals of multicellular magnetotactic prokaryotes (MMPs) in opposing oxygen gradients and magnetic fields. (*a*) Phase contrast image of MMPs, which (*b*) form a lattice at the air–water interface when the magnetic field 𝑩 is parallel to the oxygen gradient. (*c*) Example trajectory traces during the escape motility. (*d*) and (*e*) Model MMP: the bacterium’s axis of swimming aligns with its magnetic moment 𝒎 , and it experiences a torque under a uniform magnetic field. (*f*) and (*g*) Histograms of the statistics, $d_{\text{jump}}$ and $t_{\text{wait}}$ , from experiment, respectively. (*h*) MMPs exhibit three phases during escape motility: the jump phase (J) and the return phase (R), in which the bacteria move at distinct (but constant) swimming speeds, and the turnaround phase (T), in which the bacteria do not move.

Magnetotactic bacteria (MTBs) are ubiquitous and diverse bacteria, usually found in sediments of marine and freshwater habitats [1]. They were first discovered by Bellini in 1963 and coined as ‘magnetosensitive bacteria’ [2,3]. In 1975, Blakemore [4] realized that their magnetic sensitivity comes from chains of intracellular membrane-bounded nanosized magnetic crystals called magnetosomes. Blakemore viewed MTBs as self-propelled magnetic dipoles, ‘swimming compass needles’, which passively align themselves with external magnetic fields, thereby guiding their intrinsic swimming direction (figure 1*d*,*e*) [5–8]. Unicellular MTBs are theorized to navigate towards their preferred microaerobic conditions via magneto-aerotaxis, or aerotaxis with the assistance of magnetic fields. Magnetic fields enhance the efficiency of aerotaxis by restricting the motility of MTBs along the field lines, reducing a three-dimensional random walk to a one-dimensional trajectory [1,9]. The Earth’s geomagnetic field provides a directional guide for MTBs through the polarity of magnetic alignments, directing them away from the air–water interface and toward microaerobic environments in soils and sediments. Moreover, in order to keep themselves around the oxic–anoxic interface (OAI), MTBs repeatedly reverse swimming directions and form an aerotactic band around the OAI [10–12].

Owing to their striking magnetic and aerotactic properties, MTBs constitute exciting model organisms of study for biological, ecological and medical sciences. Their potential applications include, for example, environmental remediation, targeted drug delivery, magnetic resonance imaging and nanorobotics [2,13]. Their colonial relatives—multicellular magnetotactic prokaryotes (MMPs)—are spherical or ellipsoidal colonies composed of tens of bacteria, with magnetosomes aligned differently in each constituent cell, and covered in cilia which serve to propel the entire colony [14–16]. As with MTBs, MMPs also possess a magnetic dipole, which facilitates alignment in a magnetic field. Because of the complexity of their biological structure compared with unicellular MTBs, it remains unclear how MMPs actuate the collective beating of surface cilia on different cells and orchestrate changes in their motility due to chemical and physical stimuli. The lack of pure cultures of MMPs makes them challenging to study [17,18]. Here, we aim to study this through the lens of magneto-aerotaxis, where the magnetic field and oxygen gradient can be independently controlled. This allows us to establish competing cues in microfluidic experiments, challenging the colonies to make navigational decisions in the face of conflicting signals. Although previous works have demonstrated the capacity of MTBs to navigate various chemical and physical cues [11,19–21], it remains unclear how cells integrate multiple sensory cues and make navigational decisions, especially when the navigational signals are contradictory.

While magneto-aerotaxis is well studied for unicellular MTBs [11,19,22], very little is known about the process in their multicellular relatives. An intriguing difference between MMPs and unicellular MTBs is their vertical distribution—MMPs are often found in anoxic zones, deeper in the water column compared with the microaerobic regions where most unicellular MTBs prefer to reside [23,24]. Simmons *et al*. [23] found an abundance of MMPs in low sulphide regions—which also happen to be below the region of significant oxygen gradient—challenging the hypothesis that oxygen concentration influences the motility of MMPs [23]. In addition, MMPs perform negative phototaxis, which could also guide colonies to anoxic zones efficiently when paired with magnetotaxis [20,21,25], further challenging the importance of magneto-aerotaxis in MMPs. Nevertheless, there is evidence of aerotaxis performed by MMPs. Colonies are known to exhibit back-and-forth excursions in their swimming called ‘escape motility’ or ‘ping-pong motility’, usually observed near the edge of a water drop with an oxygenated boundary [15,16,18,26,27]. Note that escape motility is not exclusive to MMPs. It is also observed in other morphotypes of MTBs; however, this is less common [28]. To date, the mechanisms underpinning escape motility are unclear; however, it seems that the presence of a magnetic field stronger than the Earth’s geomagnetic field is essential [15,26,27]. The ability to perform magnetotaxis is important—no escape motility is observed in non-magnetic MMP-like aggregates (nMMPs) [25]. Our experiments, involving suspensions of MMPs near air–water and oil–water interfaces, address the essential role that oxygen gradients play in facilitating the distinctive shuttling motion, reveal that the escape motility is underpinned by a rapid and coordinated transition in the motility mode of the colonial bacterium, and demonstrate that hydrodynamic interactions between colonies play a significant role in the collective dynamics of these bacteria.

We propose an agent-based mathematical model, which allows us to investigate individual dynamics of escape motility. We start with a minimal model, in which bacteria are considered as point particles, highlighting the underlying stochastic processes. From there, near-field hydrodynamic interactions between motile colonies are included, providing a versatile model that incorporates the effects of external magnetic torques, sensing of oxygen profiles by the cells and varying colony densities and swimming modes. Mathematical models compared with the experiments illustrate that escape motility can be the consequence of a simple aerotactic mechanism.

## 2. Experiments

Fresh colonies of MMPs were collected from sediments near Palo Alto Airport, Palo Alto, CA, USA. We specifically focused on deep, anoxic regions, collecting sediment in a large centrifuge tube. These were transported to the lab and bubbled with nitrogen to maintain anoxic conditions. Although the storage was not axenic, by maintaining the full sediment core, we were able to ensure stable cultures for several months. When performing experiments, we removed a small portion of the sediment and used a small bar magnet to attract MMPs to the surface of the sample before carefully removing it. This minimized the prospect of cell contamination in the experiments performed, the success of which is confirmed visually in our reported videos. The media used in experiments consisted of filtered water from the original samples. Due to the widely reported difficulties in maintaining pure cultures of multicellular MMPs, the taxonomic details are largely undescribed. However, the morphology and behaviour are consistent with reports of other MMPs, for example, *Candidatus* Magnetoglobus multicellularis [29] and *Ca.* Magnetomorum litorale [30].

Suspensions of MMPs (mean radius = 4.3±0.5 µm, n=92 ) were extracted and placed in a hanging drop assay, with the free surface exposed to atmospheric oxygen conditions. The drop was sufficiently small to ensure the MMPs were situated in a quiescent fluid. A small permanent magnet was placed nearby so that the magnetic field lines were normal to the air–water interface (see figure 1*a*–*c*). The magnetic field was chosen to be significantly stronger than the Earth’s magnetic field, which therefore restricted the motion of MMPs to one spatial dimension. Phase contrast imaging (Nikon Ti2 with Apo TIRF 60× Oil DIC N2, 1.5× multiplier) was used to record movies of MMP motility near the air–water interface (30 fps, Photometrics Prime 95B). We observed the accumulation of MMPs near the air–water interface, guided by the direction of the magnetic field. However, individual colonies would repeatedly reverse direction, ‘jumping’ away from the interface, before changing motility mode again to return to the side of the droplet (electronic supplementary material, movies 1–3). This repeated shuttling of colonies resulted in a dynamic equilibrium (see snapshot in figure 1*b*). We repeated experiments in which the anoxic droplet was suspended within an oil droplet (electronic supplementary material, movie 4). This eliminated the presence of ‘escape jumps’, confirming the role that oxygen plays in mediating this behavioural response.

For the motility near the air–water interface, we measured the average distance $d_{\text{jump}}$ an MMP swims away from the interface (figure 1*f*) and the average time $t_{\text{wait}}$ an MMP resides in the lattice at the interface before it jumps away (figure 1*g*), taken over the span of experiment for each individual bacterium. These two statistics capture the key features of escape motility as the colonies’ movements are confined to one dimension by a strong magnetic field. From the bacterial trajectories, three phases of the escape motility were identified: jump (J), turnaround (T) and return (R). In each phase, MMPs display distinct swimming behaviour (figure 1*h*). Similar observations were made previously [27] but focused on the effect of magnetic field strength on each phase.

In separate experiments, high-speed imaging ( 1000 fps) revealed that the motility of freely swimming MMPs is characterized by axial spinning, akin to the swirling locomotion of the colonial alga *Volvox carteri* [31]. Underpinning the reversals in the swimming direction of MMP colonies is an abrupt, but highly coordinated, change in the ciliary beating on the surface. Colonies are capable of exhibiting escape manoeuvres, in which they override the magnetic signal to depart from regions of high O_ ${,}_{2}$_ concentration.

## 3. Model outline

We now turn to a model for self-propelled MMPs, capable of responding to both magnetic fields and oxygen gradients. To first examine the independent behaviour of isolated MMPs, we neglect hydrodynamic interactions and consider a colony as a self-propelled sphere with radius, a , and orientation, 𝒑 , travelling at a constant speed, $\tilde{v}$ . The bacterium’s magnetic moment, 𝒎 , is in the direction of 𝒑 , making an angle, ϕ , with the positive x -axis. Here, we assume that the MMPs are north seekers without loss of generality. Owing to the size of the colonies, they experience low Reynolds number physics (Re ∼0.001 ), and inertial forces are dominated by viscous forces. We assume zero Reynolds number in our model such that colonies always reach terminal velocities instantaneously due to the strong viscous damping, and they always experience zero net force and net torque [32]. Under a uniform magnetic field, 𝑩 , and an oxygen field, the equations governing the colony motility are given by


(3.1) $$\left\{ \begin{array}{l} \frac{d\phi }{dt}\;=\;k\;\sin\;(\phi_{B}-\phi )+\sqrt{2D_{r}}\xi (t),\\ \frac{dx}{dt}\;=\;\tilde{v}\;\cos\;(\phi )+\sqrt{2D}\xi (t),\\ \frac{dy}{dt}\;=\;\tilde{v}\;\sin\;(\phi )+\sqrt{2D}\xi (t), \end{array}\right.$$

where $k\;=\;1/\tau_{B}$ is inverse of the magnetic reaction time scale $\tau_{B}\;=\;8\pi \eta a^{3}/(\|m\|\|B\|)$ for an MMP to align its orientation with the background magnetic field, and $1/8\pi \eta a^{3}$ is the rotational mobility of a spherical particle in a fluid with viscosity η . The direction of 𝑩 makes an angle $\phi_{B}$ with the positive x -axis, and the angle between 𝒎 and 𝑩 gives rise to a torque on the bacterium, balanced by the hydrodynamic torque under the zero Reynolds number assumption. Note that we model the MMPs as points in equation (3.1); the radius a is not used to model the finite size of colonies but is used to prescribe their mobility. Magnetotactic prokaryotes perform aerotaxis by sudden reversal of their swimming direction, either parallel ( $\tilde{v}\;=\;v_{\text{jump}}$ ) or antiparallel ( $\tilde{v}\;=\;-v_{\text{return}}$ ) to their orientation with different travelling speeds (figure 1*h*). Brownian motion is modelled by the Wiener process ξ(t) with ξ(t+Δt)−ξ(t)∼N(0,1/Δt) , which describes uncorrelated noise. D and $D_{r}$ are the translational and rotational diffusion coefficients, respectively.

To model the underlying process, we assign a time-varying switching rate λ(t) to each bacterium, which controls the stochastic process of back-and-forth movements. The switching rate takes one of two values: either the basal rate, $\lambda_{-}$ , or the increased rate, $\lambda_{+}$ . A bacterium constantly updates its switching rate, λ(t) , based on its surrounding environment through a decision function, f , and waits for time exp⁡(1/λ(t)) before it reverses its swimming direction. Similar agent-based modelling was used to simulate aerotactic bands [33]; however, they did not consider escape motility. When this modelling approach is used to study escape motility, the underlying stochastic process is similar to a two-state continuous-time Markov chain (CTMC) [34], the only difference here being that we have a time-dependent parameter, λ(t) , instead of a constant, λ . If a reversal event happens, $\tilde{v}$ will be updated, from $v_{\text{jump}}$ to $-v_{\text{return}}$ and vice versa. The bipolar mechanism is used to model MMPs [11] since it is a minimal magneto-aerotaxis model in which the direction of the magnetic field plays an important role in swimming, evident by the aggregation of colonies at the air–water interface. With this choice, MMPs integrate the information from the oxygen field and the magnetic field through the following decision function:


(3.2) $$\begin{array}{r} f(c(t),\;d(t))\;=\;\left\{ \begin{array}{ll} \lambda_{+}, & \text{if (}c(t)\geq c^{*}\text{ and }d=-1\text{)}\\ & \text{or if (}c(t)<c^{*}\text{ and }d=1\text{)}\\ \lambda_{-}, & \text{otherwise}. \end{array}\right. \end{array}$$

The bacteria will reorient frequently if they sense high oxygen concentration and swim against the direction of the magnetic field or if they sense low oxygen concentration and swim along the direction of 𝑩 . The function, c(t) , is the concentration experienced by an MMP at time t , with a threshold concentration given by $c^{*}\;=\;2.5$ µM [11]. We neglect noise in the detection of oxygen concentration. The directional indicator


(3.3) $$\begin{array}{r} d(p(t),\tilde{v}(t),B)\;=\;\left\{ \begin{array}{ll} 1, & \tilde{v}p\cdot B\geq 0\\ -1, & \tilde{v}p\cdot B<0 \end{array}\right. \end{array}$$

classifies whether an MMP is swimming along or against the direction of the background magnetic field. In line with experiments, the background oxygen profile is taken to be the solution of the one-dimensional diffusion equation with boundary condition $C(0,t)\;=\;C_{0}$


(3.4) $$C(x,t)\;=\;C_{0}\;\text{erfc}\;(\frac{x}{2\sqrt{D_{C}t}}),$$

where $D_{C}$ is the diffusion coefficient of oxygen in water.

We ran a simulation with 150 MMPs initially placed at x=0 , oriented toward the left (in the direction of the interface). Oxygen starts to diffuse in for t>0 as prescribed in equation (3.4). We fitted $\phi_{B}\;=\;2.7\;\text{rad}$ , $v_{\text{jump}}\;=\;26.6$ µm s^−1^ and $v_{\text{return}}\;=\;7.6$ µm s^−1^ by taking the averages of escape jump directions, and jump and return speeds of particles from the experiment, respectively.

**Figure 2.**
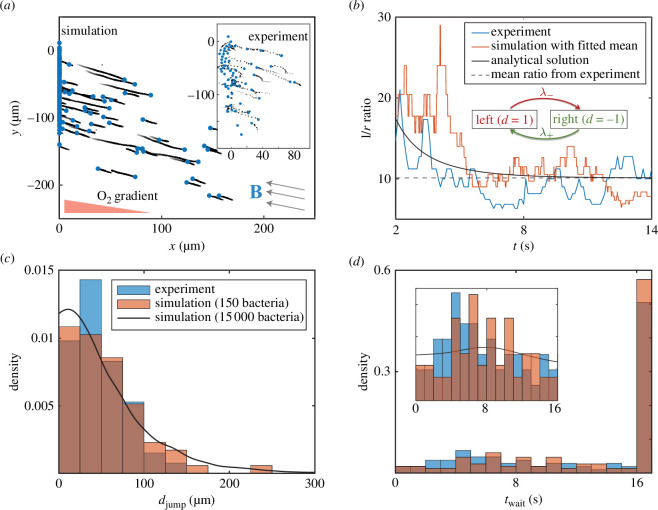
Comparison between experiments and simulations. (*a*) Simulation snapshot of 150 MMPs at t=17 s with their trajectory trails from the preceding 2 s. $\lambda_{\pm }$ , $\phi_{B}$ and $\tilde{v}$ are fitted from the experiment. Inset shows a snapshot at t=17 s from the experiment. (*b*) The ratio of a number of left swimmers and right swimmers converges in accordance with the continuous-time Markov chain (CTMC) model (equation (3.7)). Simulations were run using the mean left : right ratio extracted from experiments. Inset diagram illustrates the two-state CTMC model. (*c*) Normalized histogram for the jump distance statistics, $d_{\text{jump}}$ (excluding bacteria that never departed the interface), extracted from the simulations and experiments. (*d*) Normalized histogram for the waiting time statistics, $t_{\text{wait}}$ , compared with experiments. Inset shows the normalized histogram for the same parameter, $t_{\text{wait}}$ , but excludes bacteria that never departed the interface.


When 𝑩 is antiparallel to the oxygen gradient, we observe the formation of an aerotactic band in the simulation, with MMPs concentrating around the threshold oxygen concentration $c^{*}$ , leading to band formation around microaerobic conditions. We observe escape motility in the simulation when 𝑩 is parallel to the oxygen gradient (figure 2*a*), quickly reaching a steady state similar to the experiment (figure 1*c*). The appearance of a steady state is not surprising. At the beginning of the simulation, all MMPs are set to have a low switching rate since the concentration at x=0 is clearly greater than $c^{*}$ and the initial orientations are toward the left. Oxygen quickly diffuses in such that most MMPs detect high levels of oxygen concentration (i.e. $c(t)\;\geq \;c^{*}$ is automatically satisfied, except for a few MMPs that manage to escape to the right). Therefore, the decision function simply becomes

(3.5) $$\begin{array}{r} f(c(t),\;d(t))\;=\;\left\{ \begin{array}{ll} \lambda_{+}, & \text{if }d=-1\\ \lambda_{-}, & \text{if }d=1, \end{array}\right. \end{array}$$

and we can approximate the system by a two-state CTMC (figure 2*b*). The generator of the CTMC is given by


(3.6) $$\begin{array}{r} Q\;=\;\left[\begin{array}{cc} -\lambda_{-} & \lambda_{-}\\ \lambda_{+} & -\lambda_{+} \end{array}\right], \end{array}$$

and the steady state distribution is given by


(3.7) $$\begin{array}{r} \pi \;=\;\left[\begin{array}{cc} \pi_{\text{left}} & \pi_{\text{right}} \end{array}\right]\;=\;\left[\begin{array}{cc} \frac{\lambda_{+}}{\lambda_{+}+\lambda_{-}} & \frac{\lambda_{-}}{\lambda_{+}+\lambda_{-}} \end{array}\right]. \end{array}$$

We expect a proportion $\lambda_{+}/(\lambda_{+}+\lambda_{-})$ of the MMPs to be left swimmers and $\lambda_{-}/(\lambda_{+}+\lambda_{-})$ to be right swimmers after the CTMC has converged. This allows us to estimate $\lambda_{+}/\lambda_{-}$ by the ratio of left swimmers and right swimmers at a steady state. We can also make an inference on $\lambda_{-}$ based on the number of bacteria that did not depart the air–water interface during the finite observation time. For any bacterium, the waiting time until it performs an escape jump follows an exponential distribution, $T∼\exp(\lambda_{-})$ . The proportion of MMPs that do not leave the air–water interface is used to estimate $\text{Pr}(T\geq t_{\text{final}})$ , with ${\hat{\lambda }}_{-}\;=\;-\log(\text{Pr}(T\geq t_{\text{final}}))/t_{\text{final}}$ . The hat notation ${\hat{\lambda }}_{-}$ denotes the statistical estimation of the parameter $\lambda_{-}$ . Combined together with the previous estimate of $\lambda_{+}/\lambda_{-}$ , we obtained ${\hat{\lambda }}_{-}=0.0413$ and ${\hat{\lambda }}_{+}=0.4186$ . Note that the CTMC converges at an exponential rate $\exp\;\left(-(\lambda_{+}+\lambda_{-})t\right)$ . This is taken into account by considering the last 5 s of experimental data to estimate $\lambda_{+}/\lambda_{-}$ , once convergence is attained. With these fitted parameters, our simulation of 150 MMPs is compared against a 17 s experimental movie, and they show good agreement (figure 2*c,d*).

## 4. Hydrodynamic interactions

The hydrodynamic disturbances of a freely swimming bacterium decay quickly, with order $u∼r^{-2}$ at most [35], where r is the distance from the bacterium. This disturbance will only be significant near the air–water interface when organisms are sufficiently close to one another. In the above model, MMPs were treated as points, neglecting hydrodynamic interactions or hardcore exclusions. These effects, however, are non-negligible in the accumulation region of high colony density near the air–water interface. We, therefore, extended the model to consider MMPs as steady, spherical squirmers, whose magnetic dipole moment direction, 𝒎 , coincides with their swimming direction, 𝒑 . The squirmer is a canonical model for the flow field generated by the ciliary propulsion of a spherical microswimmer. First developed by Lighthill in 1952 [36], the squirmer model has since been extended and successfully used to model ciliary swimming, for example, of *V. carteri* [31,37]. In dense arrays of microorganisms, hydrodynamic interactions are dominated by lubrication and steric interactions, and we adapt previous numerical schemes for simulating dense arrays of these colonies [38,39]. The magnetic torque, τ=m×B , represents an equivalent effect as gravitational torque for the celebrated gravitactic squirmers [31]. For all simulations in this article, we assumed that the squirmers are pullers with squirming parameter β=1 [38,39]. We approximate the hydrodynamic interaction between an MMP and the air–water interface by the interaction of an MMP with a rigid wall. Since the colonies are enforced by the strong magnetic field to orient perpendicular to the air–water interface, the differences our approximation makes are minimal. Numerical simulations involving other values of β demonstrated qualitatively similar results and are omitted here for brevity.

**Figure 3.**
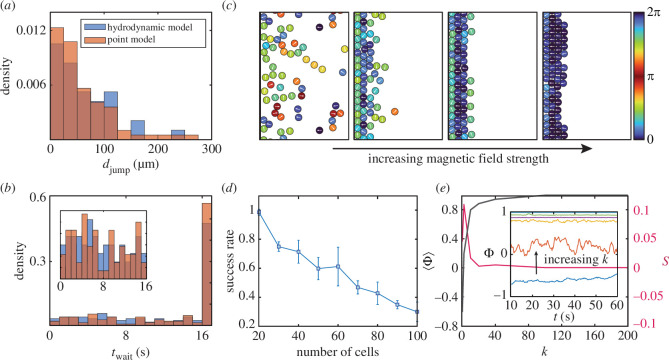
Numerical simulations of finite-sized spherical squirmers with hydrodynamic interactions. (*a*) and (*b*) Comparison of $d_{\text{jump}}$ and $t_{\text{wait}}$ between the point model and the hydrodynamic model. The packing fraction in the hydrodynamic-inclusive simulation is set to be similar to the experiment. (*c*) Simulation of MMP configurations near the boundary for different magnetic field strengths without aerotaxis. The colour bar represents the angle between the squirmer’s orientation and the magnetic field. (*d*) Rate of successful escape jumps as a function of the number of colonies, where thickness of the lattice structure grows with the colony number. A strong lattice structure was enforced by setting $k=1/\tau_{B}∼50$ s^−1^. (*e*) ⟨Φ⟩ and s as a function of k . For small magnetic field strength, there is an interplay between the hydrodynamic interactions and the magnetic alignment, and the maximum standard deviation, s , is achieved for a non-zero small k value. The inset shows the time series of Φ for the last 50 s in the simulation, which clearly demonstrates the non-monotonicity in the variance of Φ .

Because colonies have a finite size in the hydrodynamic model, they form a lattice structure near the air–water interface if the magnetic field is strong enough (figure 3*c*, last three boxes). Although each colony independently measures the oxygen profile and may attempt to reverse direction, the dense packing of colonies may prevent a successful escape jump from the lattice even if a colony has switched its swimming direction. From the simulation of the hydrodynamic model with parameters fitted from experiments, we discovered that only 66% of the attempts to escape were successful. In a regular lattice structure enforced by a strong magnetic field, the success rate in escape jumps decreases as the height of the lattice increases (figure 3*d*). The height of the lattice is measured by the number of colonies, as we initially place them in a n×10 hexagonal array [38] such that the lattice has approximately n layers throughout the simulation. When the lattice has eight layers, only approximately40% of the escape jumps are successful, which means 60% of the escape jumps could not pass 16 colony radii away from the air–water interface (defined to be roughly at the edge of the packed structure). This is a significant drop in $d_{\text{jump}}$ compared with a two-layer lattice, in which only 31% of the escape jumps could not pass 16 colony radii away from the air–water interface. Quite interestingly, the success rate when our initial condition is a 1×10 array, is approximately 80% (not shown in figure 3*d*), lower than the two-layer lattice. It is not entirely clear why this happens, but under this initial placement, we found colonies to be spaced out at the interface, and it takes longer for them to depart even after they have switched their swimming direction. We suspect that hydrodynamic interactions between side-by-side colonies can prevent escape, even if they are not directly blocked. For colonies that do successfully escape the lattice structure, colliding left- and right-swimming colonies interact hydrodynamically, but the disturbance to their trajectories is minimal in this case [40].

For the purpose of directly comparing the hydrodynamic model with the experiment and the point model, we excluded the failed attempts in the calculation of statistics. This is in line with the difficulties of experimentally identifying a failed escape jump and consistent with our data collection method. According to our simulations, the two models agree well in the ratio of left to right swimming colonies, $d_{\text{jump}}$ and $t_{\text{wait}}$ statistics (figure 3*a*,*b*), indicating our simple point model captures the observable statistics well. The good agreement is expected, as we have carefully set up the experiment under strong confinement with the magnetic field, and the dominating factor for the dynamics comes from the underlying decision process in colony motility.

A lower level of orientational order is observed in the hydrodynamic model as the magnetic field strength is weakened. This indicates an interplay between hydrodynamic interactions and magnetic field strength. To quantitatively study this, we define an order parameter $\Phi (t)\;=\;\frac{1}{N}\sum_{i=1}^{N}\cos(\phi_{i}-\phi_{B})$ to measure the average colony alignment with the magnetic field at time t . We also define *s* as the standard deviation of the time-dependent order parameter. We simulated 100 colonies with initially uniform alignment, Φ(0)=1 , and hexagonal placement near the boundary. The simulations were run for various values of k over a duration of 60 s, with aerotaxis turned off in order to fully investigate the effect of hydrodynamic interactions. The time-averaged magnetic alignment, ⟨Φ⟩ , taken over the last 50 s after Φ has stabilized, is plotted in figure 3*e*, together with the corresponding standard deviation, s , from the simulations. When k=0 , the colonies quickly disperse, and ⟨Φ⟩ falls below zero within 10 s. Dispersal also appears for weak magnetic fields until the field strength is strong enough to hold the colonies close to the boundary (figure 3*c*; electronic supplementary material, movies 5 and 6). In addition, the standard deviation, s , is a non-monotonic function of k , reaching a peak at $k\approx 2\;{\text{s}}^{-1}$ . This value, where torques due to hydrodynamic interactions are commensurate in strength to magnetic torques, marks a transition from dispersal to condensation. Similar behaviour has been recently reported by Petroff *et al*. [41], who experimentally observed the transition from an active gas to a two-dimensional active fluid upon increasing the external field strength. Because the domain in our study is two dimensional and the configurations have fewer degrees of freedom, orientation disturbances from particle–particle interactions are more pronounced in our model compared with the one reported by Petroff *et al*. [41]. Nevertheless, the phase of active matter in both cases is controlled by the competition between orientational alignment caused by the magnetic field and orientation disturbances due to hydrodynamic interactions. Since hydrodynamic disturbances decay quickly with increasing distance, the perturbations induced by hydrodynamic interactions are short-ranged and depend heavily on the configurations of colonies and boundaries.

## 5. Discussion

We experimentally observed the escape motility of multicellular MMPs near air–water interfaces. Control experiments involving immersion in oil (instead of air) highlight the essential role of oxygen in understanding escape motility. Here, we established a stochastic model based on magneto-aerotaxis and obtained good agreement with experiment. The model demonstrates that escape motility can be interpreted through a simple aerotaxis mechanism, which is a consequence of MMPs trying to integrate two competing sensory cues. In order to investigate the dense aggregation of MMPs at the air–water interface and determine how MMPs navigate more complex environments, we implemented aerotaxis in a dense array of spherical microorganisms, subject to near-field hydrodynamic and steric interactions. The hydrodynamic model unveiled interesting dynamics of the MMPs, undergoing a transition from dispersal (gas-like) to condensation (solid-like) as the magnetic field strength is increased. This elucidates an interplay between aerotaxis, magnetic alignment and hydrodynamic interactions, all of which cause reorientation of colonies and together give rise to complex dynamics. Collective dynamics in suspensions of unicellular MTBs was also observed, and in lower cell densities, far-field singularity solutions were sufficient to account for the collective instability in confined geometries [42]. However, here, the dense aggregation of cells requires resolution of the near-field hydrodynamics.

In the experimental movies, cells can appear to overlap briefly, an artefact of projecting a three-dimensional system on to two dimensions. However, despite the three dimensionality of the experimental system and, therefore, the capacity for MMPs to slide past one another in multiple layers, our two-dimensional model captures the key features of lattice stability as observed in experiments.

Previous literature reported a dependence of escape motility parameters on the magnetic field strength [26,27]. Magnetic field strength-dependent escape rates could be readily incorporated in the present model by considering the switching rate, λ , to be a function of magnetic field strength. Such a dependence was reported by Greenberg *et al*. [26], but a recent experimental study [27] replicated the experiments and found no clear changes in escape rates as the magnetic field strength varied.

Magnetotactic prokaryotes are unique magnetotactic organisms that remain multicellular in their entire life cycle [15,20,43]. Either spherical or spheroidal, their morphology facilitates convenient mathematical analysis using the squirmer model. However, the precise mechanisms by which constituent cells in an individual MMP colony coordinate to propel the whole unit remain unclear. More knowledge on this requires combined efforts in experiments, modelling and structural analysis of colonies. Colonies are known to swim in helical trajectories with body rotation [15–17]. Although under a strong magnetic field, the mean direction is well aligned to the field lines [17], additional modelling could investigate non-axisymmetric swimming (e.g. a chiral squirmer [44]). Finally, an exciting area of future research involves the investigation of MMPs interacting with complex boundaries and confined geometries. This would shed further light on the behaviour of MMPs in sediments and porous media, domains in which these intriguing organisms are found.

## Data Availability

Supporting data are available in the electronic supplementary material [45].
